# In silico receptor binding and ex vivo nasal epithelial membrane permeation studies of selected phytocannabinoids

**DOI:** 10.1186/s42238-025-00363-y

**Published:** 2026-04-16

**Authors:** Suzanne E. Van Niekerk, Theunis Cloete, Frank Van der Kooy, Josias H. Hamman

**Affiliations:** Centre of Excellence for Pharmaceutical Sciences (Pharmacen™), North-West University, Private Bag X6001, Potchefstroom, 2520 South Africa

**Keywords:** Migraine, *Cannabis sativa*, Cannabinoid receptor type 2, Receptor docking, Computer-aided drug design, Ex vivo permeation, Intranasal drug delivery

## Abstract

**Background:**

Headache disorders, specifically migraine headaches, are highly debilitating neurological disorders, with the potential to incapacitate an individual for several hours. Cannabinoid receptors are present in both peripheral and central nervous tissue, which serve as a potential target for the treatment of migraine. *Cannabis sativa* is a medicinal plant that has been used as self-medication for the treatment of headaches, but insufficient scientific information is currently available regarding their interactions with receptors, as well as intranasal delivery. The intranasal route of administration offers the potential for systemic delivery, as well as delivery into the brain. Nose-to-brain delivery offers a pathway directly to the brain via olfactory epithelium and trigeminal nerves and bypasses both the first-pass metabolism and the blood-brain-barrier (BBB).

**Methods:**

Known phytochemicals of *C. sativa* were docked in silico into the active site of the 6KPC crystal structure of the cannabinoid type 2 (CB2) receptor to screen for receptor affinity. Ex vivo permeation studies were done on these four selected cannabinoid compounds across excised sheep nasal epithelial tissue.

**Results:**

Four cannabinoid compounds were identified with affinity for the CB2 receptor that may provide activity against migraine, namely cannabicyclol, cannabidiolic acid, cannabicitran and cannabielsoin. The ex vivo membrane permeation results revealed that some of the cannabinoids can be delivered to a similar extent than moderately permeable model drugs across nasal epithelium for systemic delivery and potentially also for direct nose-to-brain delivery.

**Conclusion:**

Through affinity for the CB2 receptor, the identified compounds have shown potential in migraine treatment. There is also potential for nose-to-brain delivery.

**Graphical abstract:**

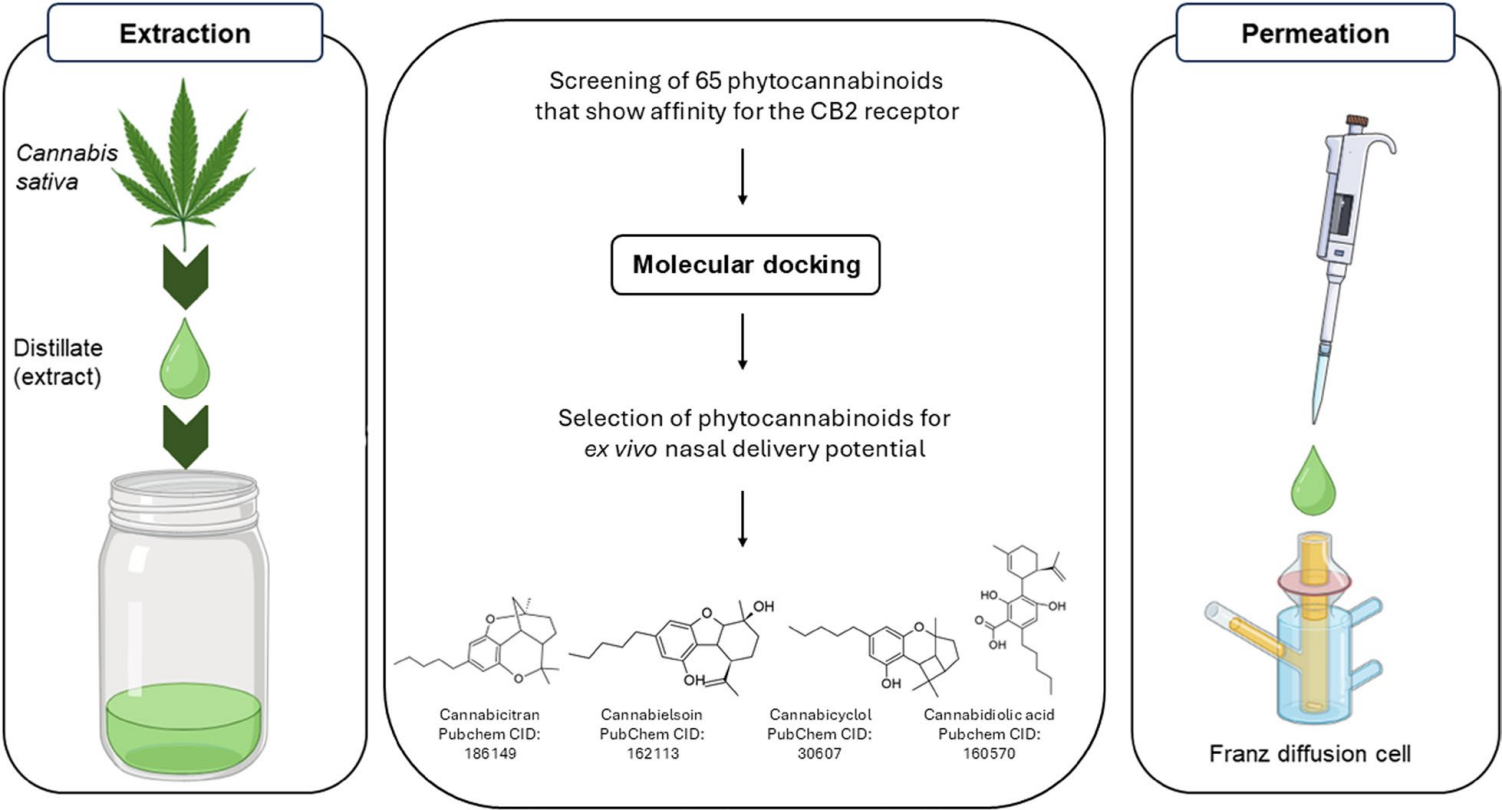

**Supplementary Information:**

The online version contains supplementary material available at 10.1186/s42238-025-00363-y.

## Introduction

Migraine is a type of headache disorder that imposes a major burden on the quality of life of patients (Martin et al. 2021). A migraine episode or attack generally occurs in phases, which consist of the pre-monitory phase, aura phase, headache phase and postdrome phase. The headache phase of a migraine attack is characterized by a severe, pulsating headache ascribed to the transfer of painful stimuli from the meninges to the cortex. The impulses are relayed by a vast network of peripheral afferent nerves to central pain structures and have connections to cortical, autonomic, and vascular structures (Qubty and Patniyot 2020). Resolution of a migraine headache is referred to as the postdrome phase. Brain homeostasis returns to normal as gradual pain relief sets in (Wolmarans de, et al., 2018).

Migraine headaches are diagnosed by at least five attacks and include a combination of the following criteria. The attack lasts 2–72 h, has a unilateral, pulsating pain of moderate to severe intensity. The pain is typically aggravated by physical activity and generally associated with nausea and vomiting, photophobia, phonophobia and osmophobia. Distinction should be made between migraine and tension headache and the severity of pain, as some symptoms overlap. There should also be no other pathology present that could cause the headache (Pryse-Phillips et al. 1997).

Migraine pathology due to both vascular and neural mechanisms has been proposed. Initially, peripheral nociceptors are activated, stimulating the release of vasoactive peptides such as calcitonin gene–related peptide (CGRP), neurokinin A, and substance P, which in turn produce an inflammatory response and elicit pain (Aurora 2004; Wolmarans et al. 2018).

It has been proposed that the endocannabinoid system could be of therapeutic value for migraine (Chandwani et al. 2023; Zorrilla et al. 2024). Two main cannabinoid receptors have been identified, namely cannabinoid type 1 (CB1) and type 2 (CB2) (Mackie 2008; Zou and Kumar 2018). CB1 receptors are distributed throughout the body, and are primarily located in the central nervous system (CNS). CB2 receptors are located to a greater extent in the immune system, however, *in situ* hybridization and immunohistochemical assays showed that CB2 receptors are present in dopamine neurons within the ventral tegmental area (Zhang et al. 2014). Although limited expression of CB2 receptors was found in the brainstem, they are more highly expressed in activated microglia during neuroinflammation (Zhang et al. 2014; Zorrilla et al. 2024). Pain pathways are sustained by over-activated microglia, releasing pro‑inflammatory mediators. Therefore, the CB2 receptor provides a potential target for migraine therapy (Sudershan et al. 2023; Sun et al. 2024).

Cannabinoids modulate neurotransmitter release via presynaptic activity, alter postsynaptic neuron excitability, activate descending inhibitory pathways and reduce neural inflammation. Activation of the signal cascade involve the inhibition of adenylyl cyclase, a decrease in cAMP formation, and an increase in mitogen-activated protein kinases (MAPK) activity (Baron 2015; Vučkovic et al. 2018).

CB1 receptor activation leads to the inhibition of GABA, glutamate, acetylcholine, serotonin, dopamine and norepinephrine. CB1 agonist receptor binding decreases presynaptic neuron firing by activating inward-rectifying potassium channels and decreases neurotransmitter release by suppressing voltage-sensitive calcium channels. CB2 receptors inhibit nociception on peripheral sensory neurons by the release of β-endorphin which activate µ-opioid receptors. CB2 receptor activation induces immunosuppression and the reduction of pro-inflammatory cytokines but calcium and potassium conductance modulation are controversial (Baron 2015; Guindon and Hohmann 2008; Vučkovic et al. 2018). The psychotropic effects of cannabinoids are mainly attributed to the activation of CB1 receptors in the CNS (Vučkovic et al. 2018). On the other hand, CB2 receptors have been identified as an attractive therapeutic target for immunomodulation, as well as the treatment of inflammation, neuropathic pain, neurodegenerative disorders and migraine, while avoiding the psychotropic side effects mediated by CB1 (Poudel et al. 2021). The less side effects and neuro-protective features associated with CB2 receptors can be attributed to the distribution of these receptors in the post-synaptic neuronal somatodendritic areas of the brain, whereas CB1 receptors are expressed on presynaptic GABAergic terminals in the ventral tegmental area (Zhang et al. 2014).

The CB2 receptor has been investigated in an animal model for pain management and for the potential as a target in the treatment of migraine (Greco et al. 2014). Hyperalgesia was induced in male Sprague-Dawley rats through systemic administration of nitroglycerin. The CB2 agonist, AM1241-o-dimethyl sulfoxide, was administered as treatment and shortly after, one of two pain models; the tail flick test or the formalin test was performed on the rats. The CB2 agonist presented with a significant analgesic effect for both tests. In addition, an anti-hyperalgesic effect was detected during phase II of the formalin test, which represents a state of hyperalgesia that is mediated by the peripheral release of inflammation mediators and central modulation. By counteracting the nitroglycerin induced hyperalgesia and flinches/shakes experienced during phase II of the formalin test, the activation of CB2 was found to be a promising alternative to CB1 activation as it is also less likely to induce central nervous system side effects (Greco et al. 2014).

The receptor mechanisms mediating anti-neuroinflammatory effects of endocannabinoid system modulation were investigated in an in vivo migraine model and *ex-vivo* hemiskull preparations in rats. Methanandamide, a cannabinoid agonist, was found to deliver CGRP modulation through CB1 receptors, and inhibition of degranulation of dural mast cells in the cranial dura mater through CB2 receptors. As neurogenic inflammation in migraine pathology can be ascribed to CGRP, substance-P and dural mast cells, ligands that target CB1 and CB2 receptors should be investigated as potential migraine therapies (Kilinc et al. 2022).

Non-selective cannabinoid receptor agonist, WIN 55,212-2, activated CB1 and CB2 receptors in a nitroglycerine-induced animal model and formalin test conducted on rats. Attenuation of migraine-like pain was reported (Vosough et al. 2019).

A survey was conducted on data from a free medical cannabis use application, Strain print, where individuals log in and track the severity and relief of their symptoms as a function of the dose and strain of cannabis used (Cuttler et al. 2020). Most individuals inhaled (smoked) either a cannabis flower or concentrated product. Use of these cannabis products resulted in an overall reduction of self-reported headache and migraine severity by approximately 50%. A few similar studies have been conducted, which also found cannabis to decrease migraine headaches (Piper et al. 2017; Rhyne et al. 2011). A systematic review found that migraine-day frequency can be lowered and nearly 12% of attacks could be prevented when using medicinal cannabis (Okusanya et al. 2022). Furthermore, a double-blind, placebo-controlled crossover trial conducted on 92 participants showed that a vaporised cannabis treatment produced 2-hour pain relief and pain-freedom (Schuster et al. (Schuster 2024)).

Since nausea and vomiting are frequently associated with migraine attacks, patients may often be reluctant to take oral treatment (Martin et al. 2021). Furthermore, it is challenging to effectively deliver drug molecules into the brain after oral administration due to the shielding function of the blood-brain barrier (BBB). Typically, only 1–5% of the bioavailable drug dose in the systemic circulation reaches the brain (Sonvico et al. 2018). The nasal route of drug administration is a promising alternative to oral and parenteral routes of administration due to its non-invasive nature to deliver drugs into the systemic circulation and/or directly into the brain. Furthermore, the nasal route of administration bypasses the BBB and first pass metabolism and delivers a short onset of action (Keller et al. 2022; Salade et al. 2019).

Therefore, this study aimed to determine which known phytochemicals of *C. sativa* presented with affinity for the CB2 receptor. Identified compounds were subjected to ex vivo permeation studies across excised sheep nasal epithelial tissue to determine whether the compounds could potentially be delivered effectively following intranasal delivery.

## Materials

Reference standards for canabigerolic acid (CBGA), cannabicyclol, cannabidiolic acid (CBDA), cannabicitran and cannabielsoin were purchased from LGC (Johannesburg, South Africa). All reference compounds had a purity of > 98%. *Cannabis sativa* distillate (cannabinoid containing extract) was generously donated by GES Labs (Cape Town, South Africa). Mass spectrometry (MS) grade acetonitrile, methanol and ultrapure water (Merck, Germany), olive oil and dimethyl sulfoxide (DMSO) was purchased from Sigma-Aldrich (South Africa).

## Methods

### In silico docking to determine affinity of cannabinoid phytochemical compounds for the CB2 receptor

**Fig. 1:**
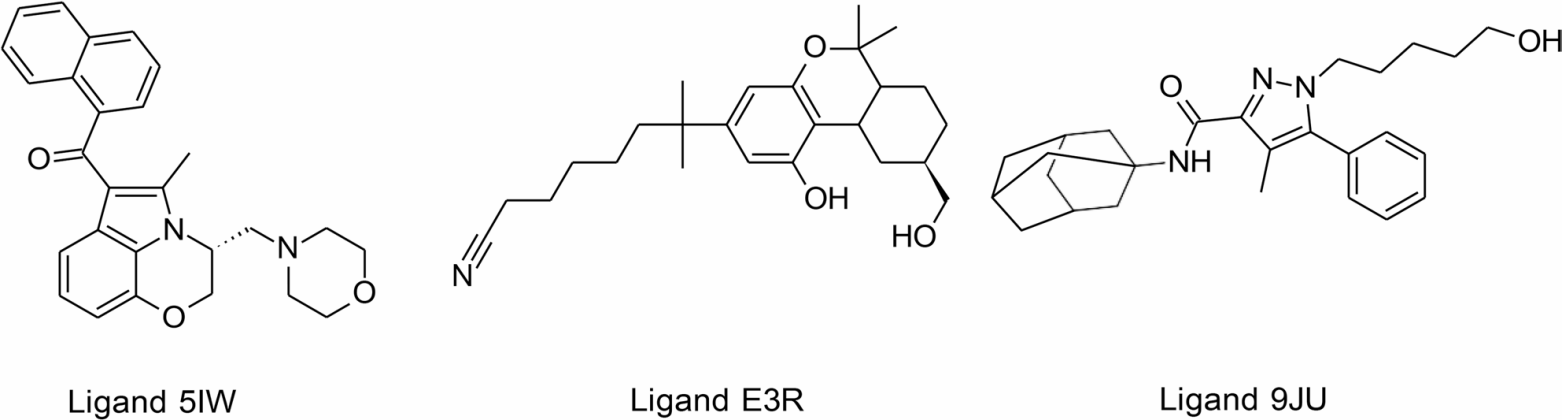
Chemical structures of the antagonist (9JU) and agonists (E3R & 5IW) that were co-crystallized within the active site of the cannabinoid type 2 (CB2) receptor

Discovery Studio V3.1 (Accelrys Inc., USA) (San Diego, United States) software was used to conduct the docking studies of cannabinoid phytochemical compounds for affinity to the CB2 receptor. Following a literature review, three CB2 receptor crystal structures were obtained from the Protein Data Bank (PDB) (Berman et al. (Berman 2000)) namely 6KPC (ligand: E3R) (Hua et al. 2020), 6PT0 (ligand: 5IW) (Xing et al. 2020) and 5ZTY (ligand: 9JU) (Li et al. 2019). Each crystal structure contains information about each atom of the receptor, including its coordinates (Maveyraud and Mourey 2020). The three CB2 receptor crystal structures contained either an antagonist (9JU) or an agonist (E3R & 5IW) that was co-crystallized within the active site (Fig. 1) (Hua et al. 2020; Li et al. 2019; Xing et al. 2020).

Unless otherwise specified, the default settings in Discovery Studio software program were selected. To prepare each receptor crystal structure, the “prepare protein protocol” was utilized. Subsequently, the CHARMm forcefield was applied and protein ionization and pK values were calculated. The protein backbone was then constrained, and the smart minimization algorithm was employed for protein minimization with a maximum step count of 50 000. The minimization process utilized the implicit generalized born with molecular volume (GBMV) solvent model, and the SHAKE constraint was not applied. Both the co-crystallized ligand and water molecules were removed after which the entire protein was identified as the CB2 receptor. The QC steps incorporated allowed for accurate identification of the ligands. Default active sites were used except if results required active site changes.

If necessary, corrections were made to the structure of each co-crystallized ligand (9JU, E3R and 5IW). Hydrogens were added to the ligands, and a brief optimization of their geometry was performed. The “prepare ligands protocol” was used to prepare all ligands.

Docking was carried out using the LigandFit and LibDock docking algorithms and the results were rescored using the LigScore 1 & 2 (Krammer et al. 2005), Piecewise Linear Potential (PLP) 1 & 2 (Verkhivker et al. 2000, Jain 2006), Potential of Mean Force (PMF) (Muegge 2006) and PMF 4 scoring functions, in addition to the native scoring function of each docking program. This resulted in eight docking/scoring combinations per docking algorithm and 16 docking/scoring combinations in total. For LigandFit, 100 randomly generated conformations were created. For LibDock, the conformation method was set to BEST, and 100 hotspots were retained. *In situ* minimization of the solutions was not performed.

#### Root-mean-square Deviation (RMSD)

RMSD success rates determine the accuracy of the experimentally observed position of a co-crystalized ligand in comparison to the prediction of the docking program. Generally, values smaller than 2 angstrom (Å) indicate successful redocking, while values smaller than 1.5 Å should be used if the ligand has a low number of rotatable bonds (Cole et al. 2005; Hevener et al. 2009).

The co-crystallized ligands, i.e., E3R, 5IW and 9JU were re-docked into the active site of 6KPC, 6PT0 and 5ZTY using all the different docking/scoring combinations. The heavy atom root-mean-square deviation (RMSD) (Å) values were calculated with Discovery Studio software program and an RMSD threshold of < 2 Å was used as a cut-off to identify compounds that were successfully redocked (Cole et al. 2005; Hevener et al. 2009; Kramer et al. 1999; Vieira et al. 2022). The success rate is an expression of the percentage of ligands that were successfully redocked and were calculated using Eq. 1 (Cole et al. 2005).

1 $$\begin{aligned}\text{Success rate}=(&\text{Number of ligands}\;<\;2\;\overset\circ{\mathrm{A}}\\&/\text{Total number of ligands})\;\times\;100\end{aligned}$$

#### Enrichment Factor (EF) and Receiver Operating Characteristic (ROC) curve

To determine whether the program can accurately identify active compounds, known active and inactive (decoy) compounds are screened by each docking/scoring combination, and the ability of the combination to correctly identify the active compounds and disregard the decoy compounds were quantified by calculating the enrichment factor (EF) and the receiver operating characteristic (ROC) curves. The EF was calculated with Microsoft Excel for (Microsoft 365, United States of America) using Eq. 2. The Schrödinger decoy set was downloaded and contains druglike decoys with a mean molecular mass of 400 Daltons (Halgren et al. 2004; Hevener et al. 2009). Three hundred decoys were prepared as previously mentioned using the prepare ligands protocol. The co-crystallized ligands, i.e., E3R, 5IW and 9JU were seeded into the decoy database and the different docking/scoring combinations were tasked to identify the active compounds within this mixed database. The EF was calculated for the top 10% of the database and is an expression of the proportion of active compounds correctly identified in the top percentage compared to the rest of the database (Chen et al. 2006; Li et al. 2019; Wei et al. 2002).

2 $$\mathrm{EF}\;=\;({\mathrm{Hits}}_\mathrm{sel}\;/\;{\mathrm{Hits}}_\mathrm{tot})\; \mathrm{X}\;(\mathrm{NC}_\mathrm{tot}\;/\;\mathrm{NC}_\mathrm{sel})\;\;[Math Processing Error]$$

Hits_sel_ refers to the number of active compounds within the top 10% of the dataset. Hits_tot_ represents the total number of active compounds within the entire database. NC_tot_ denotes the total number of compounds (active and decoy) in the entire database, while NCsel represents the number of compounds (active and decoy) within the selected percentage, i.e., 10% (Chen et al. 2006; Wei et al. 2002).

Subsequently, the ROC curves were constructed using SPSS Statistics for Windows, version 27 (IBM, United States). The area under the ROC curve (ROC-AUC) is a practical means of interpreting the results to evaluate the overall performance of the screening experiment (Lätti et al.^ 2016^; Triballeau et al. 2005). A ROC-AUC of 0.9 ≤ AUC ≤ 1.0 is considered excellent, between 0.8 ≤ AUC < 0.9 is considered good, between 0.7 ≤ AUC < 0.8 is considered fair, between 0.5 ≤ AUC < 0.7 is considered poor and below 0.5 is considered a failure (Braga and Andrade 2013).

### Virtual ligand screening

Phytochemicals commonly found in *C. sativa* were identified by means of a literature search (Baron 2018; El-Shenawy et al. 2021; Turner et al. 1980) and their chemical structures were downloaded from the PubChem database and prepared as described above using the “prepare ligands protocol” (Kim et al. 2023).

All compounds were docked with both the LigandFit and LibDock docking algorithms into the active site of the crystal structure and rescored with scoring functions that have been determined to deliver accurate results (as described above). The *C. sativa* phytochemical compounds that were identified to have affinity for the active site of the CB2 receptor by either the LigandFit or LibDock docking algorithms and with any of the validated scoring functions were recorded and cannabinoids that could be obtained were included in the nasal membrane permeation studies.

### Liquid Chromatography Mass Spectroscopy (LC-MS) and fluorometric analytical methods

For this study, different model drugs with varying membrane permeation properties were selected to serve as reference compounds that could be used as a benchmark for the evaluation of the membrane permeation of the selected cannabinoids after application of a cannabinoid extract. The following model compounds were selected namely Lucifer Yellow (LY) (exclusion marker molecule with extremely low membrane permeability), acyclovir (low membrane permeability) and atenolol (moderate to high membrane permeability) (FDA 2017).

LC-MS analytical methods for the selected cannabinoid compounds and model drugs were developed and subsequently validated (ICH (ICH. 2005)). Sample analysis was conducted on an Agilent Ultivo^®^ triple quadrupole mass spectrometer fitted with an Agilent^®^ 1260 Infinity quaternary pump, autosampler and column heater. The LC-MS method parameters are given in Table 1.

All the analytical methods were validated by preparing a serial dilution for each compound/model drug ranging from 0.19 to 50.00 µg/mL for LY; 0.98–500.00 ng/mL for model drugs (acyclovir and atenolol); 0.98–500.00 ng/mL for CBDA, cannabicyclol and cannabicitran and 0.098–100.000 ng/mL for cannabielsoin. Working range and linearity were established with a correlation coefficient (R^2^) of ≥ 0.98. Precision was determined for the newly developed LC-MS methods by intra-day and inter-day repeatability, an in-lab maximum relative standard deviation of 11% or less was considered acceptable. The limit of detection and limit of quantification were determined for each analyte and working ranges were set-up accordingly. All collected data points were analyzed and processed above the respective limits of quantification (Rambla-Alegre et al. 2012).

LY was analyzed by means of fluorescence spectroscopy using a Spectramax^®^ Paradigm plate reader with excitation and emission wavelengths pre-set to 485 nm and 535 nm, respectively (Rozehnal et al. 2012).

**Table 1:** Instrument parameters for LC-MS analytical methods

*Cannabis sativa compounds*	LC Conditions	Solvents	Solvent Ratio (%)	Flow (mL/min)	Total run time (min)	Diverter valve to MS at time (min)	Retention time (min)	Column
	Cannabielsoin	Water + 0.1% FA MeOH + 0.1% FA	20 80	0.250	6.0	1.0	2	Phenomenex Kinetex 2.6 μm F5 100 Å 100 × 2.1 mm
	CBDA						2.3	
	CBGA						3	
	Cannabicyclol						3.7	
	Cannabicitran						5	
	MS Conditions	Precursor ion (m/z)	Product ion (m/z)	Fragmentor Voltage (V)	Polarity			
	Cannabielsoin	331.2	205.2/107.0	86	+			
	CBDA	359.6	341.2/114.7	71	+			
	CBGA	359.2	191.0/341.1	96	+			
	Cannabicyclol	315.2	235.1/234.6	91	+			
	Cannabicitran	315.2	259.1/123.1	96	+			
Model Drugs	LC Conditions	Solvents	Solvent Ratio (%)	Flow (mL/min)	Total run time (min)	Diverter valve to MS at time (min)	Retention time (min)	Column
	Acyclovir	Water + 0.1% FA	85	0.350	4.0	1.5	2.8	Phenomenex Kinetex 2.6 μm F5 100 Å 100 × 2.1 mm
		MeOH + 0.1% FA	15					
	Atenolol	Water + 0.1% FA	60	0.300	3.0	1.0	1.8	
		ACN + 0.1% FA	40					
	MS Conditions	Precursor ion (m/z)	Product ion (m/z)	Fragmentor Voltage (V)	Polarity			
	Acyclovir	226.1	152.0/135.1	61	+			
	Atenolol	267.2	116.0/107.0	96	+			

### Selected cannabinoid compound content assay of extract

The *C. sativa* cannabinoid containing extract was assayed to determine the percentage content of each of the selected individual cannabinoid compounds, namely CBGA, CBDA, cannabielsoin, cannabicyclol and cannabicitran. 

### Sheep nasal epithelial tissue collection and preparation

The research project was approved by the North-West University Animal Care, Health and Safety Research Ethics Committee (NWU AnimCare, with approval nr NWU-00439-21-A5) for use of excised tissues from animals already slaughtered at an abattoir and therefore there were no study-related animal welfare implications. Nasal respiratory epithelial tissue was excised from a sheep directly after slaughtering the animal at the local abattoir using a method previously described (Du et al. 2006; Gerber 2022). Firstly, the top, front section of the snout was removed with a transverse incision anterior to the eyes. The snout was immediately rinsed with and subsequently immersed in ice-cold Krebs Ringer bicarbonate buffer solution and transported to the laboratory.

In the laboratory, the tip of the snout was removed, and the snout was cut longitudinally along the septal midline with a Mac Afric^®^ bone saw into two halves (Garyton Industry Co. LTD, Zhejiang, China). Incisions were made perpendicular along the ventral nasal concha to facilitate the removal of the respiratory epithelium from the cartilage. The detached epithelial tissue was dissected into smaller sections of approximately 15 × 15 mm and mounted onto the Franz diffusion half-cells.

### Membrane integrity test

The integrity of the excised sheep nasal respiratory epithelial tissue mounted in the Franz diffusion cells was verified by performing a membrane permeation study with the marker molecule, LY. A volume of 1 mL of a LY solution (50 µg/mL) was added to each apical chamber of 6 Franz diffusion cells and 2 mL KRBB containing 0.5% v/v DMSO in the basolateral chambers. The total volume (2 mL) was withdrawn from the acceptor chambers and subsequently replenished with pre-heated (37 °C) KRBB at pre-determined time intervals. The water bath containing the 6 diffusion cells was kept at 37 °C throughout the permeation study. 200 µL of each sample was transferred to the wells of a Costar^®^ 96-well plate and then quantified by fluorescence spectrophotometry (SpectraMax^®^ Paradigm plate reader) at an excitation wavelength of 485 nm and an emission wavelength of 535 nm (Rozehnal et al. 2012).

### Cannabinoid and model compound permeation across excised nasal epithelial tissue

Permeation studies were performed by placing 1 mL each of the aqueous model drug solutions (LY solution at 50 µg/mL; acyclovir and atenolol solutions at 40 µg/mL), as well as the *C. sativa* cannabinoid containing extract dissolved in olive oil (20 mg/mL solution) in the apical chambers of 6 Franz diffusion cells and 2 mL KRBB containing 0.5% v/v DMSO in the basolateral chambers under continuous magnetic stirring. The total volume (2 mL) of the basolateral chamber was withdrawn at pre-determined time intervals namely 20, 40, 60, 80, 100 and 120 min. Following each withdrawal, the basolateral chamber was replenished with 2 mL preheated KRBB containing 0.5% v/v DMSO. All studies were completed with the water bath temperature set to 37 °C. After the permeation studies were completed, tissue samples were lysed by cutting the tissue into smaller segments, adding 5 mL methanol, sonicating the samples for 10 min and then centrifuging the samples at 3000 rpm for 5 min. All the permeation studies were conducted with 6 replicates (*n* = 6). The samples were analyzed by means of the LC-MS or fluorometric analytical methods as previously described and the mean apparent permeability (P_app_) (Sousa and Castro (Sousa, and Castro, 2016)), percentage transport and the amount of phytochemical in the tissue membrane was calculated for each group.

## Results

### Preparation of CB2 ligands

For both the 6KPC and 5ZTY crystal structures, the active site was correctly identified by the Discovery Studio software program as site 1, while for 6PT0, the active site was identified as site 2. The default active site size was used except for 6PT0 and 5ZTY when docking with LigandFit, where the active site size was reduced to 16.1 Å (6PT0) and 15.1 Å (5ZTY) to obtain more reliable results.

### In silico docking to determine affinity of cannabinoid phytochemical compounds for CB2 receptor

In silico docking consists of docking and scoring, where the docking algorithm first docks different poses of a ligand into the active site, after which the scoring function identifies the most favorable pose and assigns different scores to each pose. Different docking algorithm and scoring function combinations can produce different results (Kroemer 2007).

This section will discuss the results of the eight docking/scoring combinations per docking algorithm and 16 docking/scoring combinations.

#### Root-mean-square Deviation (RMSD)

**Fig. 2:**
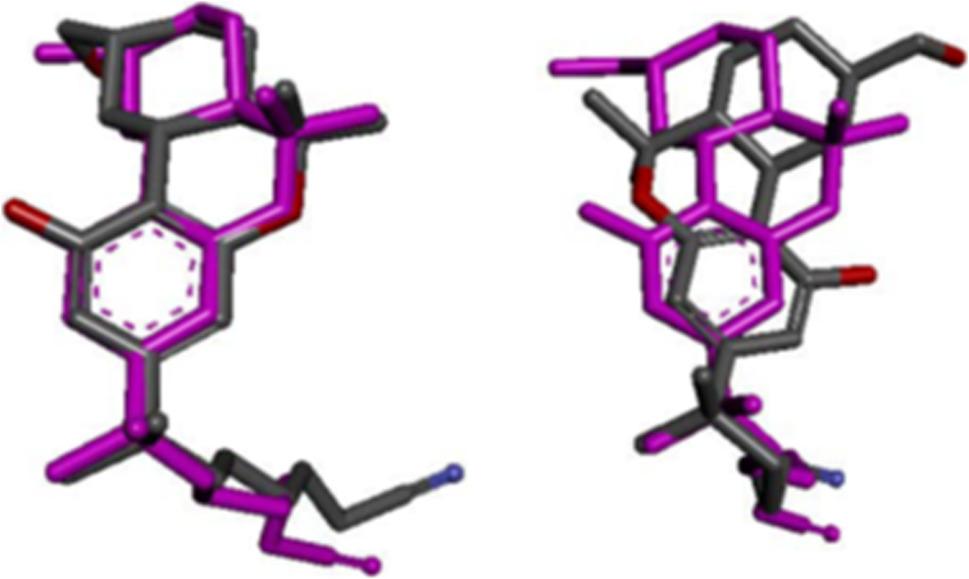
Schematic illustration of the difference between the co-crystallized ligand, i.e., E3R, that has been re-docked acceptably (RMSD: 0.8 Å) (left) vs. re-docked not acceptably (RMSD: 3.3 Å) (right). The original co-crystallized ligand is indicated in purple

The RMSD is a parameter used to measure the distance in Å between the atoms of the original co-crystallized ligand and that same co-crystallized ligand after it has been re-docked (Bell and Zhang 2019). An RMSD cut-off value is used to differentiate between a ligand that has been re-docked successfully (< 2 Å) or unsuccessfully (≥ 2 Å) (Fig. 2) (Cole et al. 2005; Hevener et al. 2009; Kramer et al. 1999; Vieira et al. 2022).

When re-docking more than one co-crystallized ligand, the percentage of ligands docked successfully is known as the success rate (Cole et al. 2005). The RMSD results of all the docking/scoring combinations after the three co-crystallized ligands (9JU, E3R, 5IW) were re-docked into the binding site of the three different crystal structures of the CB2 receptor are shown in Table 2, below. Information regarding the three ligands is displayed in the ligands’ column with the total number of poses generated for each ligand indicated in brackets. The best pose column indicates the lowest RMSD values considering all the poses with the pose rank indicated in brackets. A pose refers to the conformation and orientation of a ligand that has been docked into the active site (Torres et al. 2019). The RMSD of the top pose identified by each scoring function, including the native scoring function for both LibDock and LigandFit, are shown in the subsequent columns each time with the pose rank displayed in brackets. Below each scoring function the average RMSD value for all three co-crystallized ligands are displayed.

**Table 2:** Root-mean-square deviation (RSMD) values of poses generated by different docking/scoring combinations for 6PT0, 6KPC and 5ZTY

Root-mean-square deviation (RSMD) values for 6PT0	Docking Program	Ligands	Best pose	Scoring functions							
		(Number of poses)	(Pose rank)	Native‡ (1st pose)	LigScore 1	LigScore 2	PLP 1	PLP 2	Jain	PMF	PMF 4
					(Pose rank)	(Pose rank)	(Pose rank)	(Pose rank)	(Pose rank)	(Pose rank)	(Pose rank)
	LibDock‡	9JU (98)	1.16 (96)	8.68	1.84 (72)	1.84 (72)	8.60 (7)	8.60 (7)	8.82 (16)	4.58 (87)	4.58 (87)
		E3R (98)	1.65 (81)	7.99	8.10 (26)	8.10 (26)	7.83 (3)	7.83 (3)	4.66 (66)	1.65 (81)	1.65 (81)
		5IW (100)	1.02 (57)	1.87	1.02 (57)	1.02 (57)	1.63 (15)	1.87 (1)	2.54 (45)	2.09 (30)	7.95 (97)
		Total*	3	1	2	2	1	1	0	1	1
		Success rate†	100%	33%	67%	67%	33%	33%	0%	33%	33%
		Average RMSD	1.3	6.2	3.7	3.7	6.0	6.1	5.3	2.8	4.7
	LigandFit+	9JU (100)	1.18 (65)	1.84	1.63 (30)	1.92 (81)	1.37 (12)	1.64 (47)	1.63 (30)	1.77 (24)	2.06 (100)
		E3R (100)	1.41 (93)	8.11	8.01 (4)	8.28 (10)	2.59 (86)	2.59 (86)	2.59 (86)	8.09 (33)	8.24 (100)
		5IW (7)	1.00 (2)	1.06	1.06 (1)	1.06 (1)	1.06 (1)	1.06 (1)	8.40 (6)	1.06 (1)	2.46 (7)
		Total*	3	2	2	2	2	2	1	2	0
		Success rate†	100%	67%	67%	67%	67%	67%	33%	67%	0%
		Average RMSD	1.2	3.7	3.6	3.8	1.7	1.8	4.2	3.6	4.3
	Overall success rate§		100%	50%	67%	67%	50%	50%	17%	50%	17%
Root-mean-square deviation (RSMD) values for 6KPC	Docking Program	Ligands	Best pose	Scoring functions							
		(Number of poses)	(Pose rank)	Native‡ (1st pose)	LigScore 1	LigScore 2	PLP 1	PLP 2	Jain	PMF	PMF 4
					(Pose rank)	(Pose rank)	(Pose rank)	(Pose rank)	(Pose rank)	(Pose rank)	(Pose rank)
	LibDock‡	9JU (98)	1.96 (34)	8.46	3.32 (15)	3.32 (15)	8.46 (1)	8.46 (1)	8.46 (1)	3.32 (15)	3.32 (15)
		E3R (98)	0.67 (41)	2.20	0.76 (15)	0.76 (15)	2.20 (1)	2.20 (1)	7.62 (73)	0.67 (41)	0.67 (41)
		5IW (100)	1.58 (49)	1.97	8.42 (58)	8.42 (58)	1.81 (3)	2.60 (23)	2.71 (94)	2.16 (18)	2.16 (18)
		Total*	3	1	1	1	1	0	0	1	1
		Success rate†	100%	33%	33%	33%	33%	0%	0%	33%	33%
		Average RMSD	1.4	4.2	4.2	4.2	4.2	4.4	6.3	2.0	2.0
	LigandFit+	9JU (100)	2.38 (42)	2.53	2.59 (10)	2.71 (47)	2.73 (76)	2.56 (59)	2.80 (15)	2.73 (77)	2.69 (53)
		E3R (100)	0.68 (57)	0.81	1.15 (49)	0.81 (1)	0.74 (14)	0.74 (14)	1.73 (96)	0.80 (17)	0.88 (51)
		5IW (7)	1.04 (54)	2.20	8.32 (51)	1.23 (22)	1.33 (3)	1.41 (6)	1.96 (33)	2.33 (2)	2.33 (2)
		Total*	2	1	1	2	2	2	2	1	1
		Success rate†	67%	33%	33%	67%	67%	67%	67%	33%	33%
		Average RMSD	1.4	1.8	4.0	1.6	1.6	1.6	2.2	2.0	2.0
	Overall success rate§		83%	33%	33%	50%	50%	33%	33%	33%	33%
Root-mean-square deviation (RSMD) values for 5ZTY	Docking Program	Ligands	Best pose	Scoring functions							
		(Number of poses)	(Pose rank)	Native‡ (1st pose)	LigScore 1	LigScore 2	PLP 1	PLP 2	Jain	PMF	PMF 4
					(Pose rank)	(Pose rank)	(Pose rank)	(Pose rank)	(Pose rank)	(Pose rank)	(Pose rank)
	LibDock‡	9JU (98)	1.46 (71)	6.98	1.97 (24)	1.97 (24)	7.21 (9)	7.21 (9)	7.30 (84)	2.18 (69)	7.21 (9)
		E3R (98)	10.72 (17)	12.05	11.54 (63)	12.18 (85)	11.20 (2)	11.20 (2)	12.78 (64)	11.94 (3)	11.94 (3)
		5IW (100)	1.27 (23)	7.72	5.27 (10)	5.27 (10)	6.60 (2)	6.60 (2)	7.79 (40)	8.05 (62)	7.94 (55)
		Total*	2	0	1	1	0	0	0	0	0
		Success rate†	67%	0%	33%	33%	0%	0%	0%	0%	0%
		Average RMSD	4.5	8.9	6.3	6.5	8.3	8.3	9.3	7.4	9.0
	LigandFit+	9JU (100)	0.85 (7)	0.97	1.18 (40)	1.19 (26)	0.97 (1)	1.07 (78)	1.51 (85)	1.20 (13)	1.20 (13)
		E3R (100)	10.92 (37)	11.88	11.91 (25)	11.94 (100)	11.77 (29)	11.77 (29)	11.85 (61)	11.59 (10)	11.60 (8)
		5IW (7)	0.72 (1)	0.72	0.72 (1)	0.72 (1)	1.39 (48)	1.39 (48)	7.93 (18)	0.72 (1)	7.88 (61)
		Total*	2	2	2	2	2	2	1	2	1
		Success rate†	67%	67%	67%	67%	67%	67%	33%	67%	33%
		Average RMSD	4.2	4.5	4.6	4.6	4.7	4.7	7.1	4.5	6.9
	Overall Success rate§		67%	33%	50%	50%	33%	33%	17%	33%	17%

* Number of ligands with an RMSD value < 2

† Percentage of ligands with and RMSD value < 2

‡ Native scoring function for LibDock (LibDock Score) & native scoring function for LigandFit (Dock Score)

Success rate of the relevant scoring function taking both docking functions into account

 The LibDock docking algorithm was able to correctly dock all three ligands into the 6PT0 CB2 receptor crystal structure, when considering the best pose column, i.e., an RMSD value of < 2 Å, resulting in a success rate of 100% (Table 2). This indicates that the LibDock docking algorithm has the ability to correctly dock the ligands into the active site. When all the scoring functions were combined with LibDock, LigScore 1 & 2 performed the best by correctly identifying two ligands with an RMSD of < 2 Å, resulting in a success rate of 67% (2/3 × 100%) and an average RMSD of 3.7 Å (Table 2). The LigandFit docking algorithm also had a success rate of 100%. Using the scoring functions together with the LigandFit docking algorithm, the native scoring function (Dock Score), LigScore 1 & 2, PLP 1 & 2 and PMF all performed equally well, each with a success rate of 67%. PLP 1 had the lowest average RMSD (1.7 Å) followed by PLP 2 (1.8 Å), then LigScore 1 and PMF (3.6 Å), Dock Score (3.7 Å) and then LigScore 2 (3.8 Å) (Table 2).

With regards to the 6KPC CB2 receptor crystal structure, the LibDock and LigandFit docking algorithms had a success rate of 100% and 67%, respectively (Table 2). The LibDock docking algorithm together with all the scoring functions, performed poorly with a maximum success rate of 33% (Table 2). PMF and PMF 4, however, each had an average RMSD of 2 Å. The LigandFit docking algorithm performed better with a success rate of 67% for the scoring functions LigScore 2, PLP 1 & 2 and Jain. LigScore 2, PLP 1 & 2 had an average RMSD of 1.6 Å and Jain had a value of 2.2 Å (Table 2).

Both the LibDock and LigandFit docking algorithms had a success rate of 67% for the 5ZTY CB2 receptor crystal structure (Table 2). When using the LibDock docking algorithm, only the LigScore 1 & 2 scoring functions were able to correctly identify a pose with an RMSD < 2 resulting in a success rate of 33%, and an average RMSD of 6.3 and 6.5 respectively (Table 2). The LigandFit docking algorithm performed better, with the Dock Score, LigScore 1 & 2, PLP 1 & 2 and PMF scoring functions all presenting with a success rate of 67%. The Dock Score and PMF scoring functions had the lowest average RMSD value (4.5 Å), followed by LigScore 1 & 2 (4.6 Å) then PLP 1 & 2 (4.7 Å) (Table 2).

In conclusion, the success rate achieved by both the LibDock and LigandFit docking algorithms indicate that they are capable of redocking the co-crystallized ligands correctly when paired with the correct scoring functions (Table 2). For the subsequent validation experiments the 6KPC crystal structure was used. This decision was based not only on the success rates achieved when using the 6KPC crystal structure but also considering the average RMSD score of each combination.

#### Enrichment Factor (EF) and Receiver Operating Characteristic (ROC) curve

The EF^10%^ is a parameter used to measure the ability of a docking program to identify active compounds within a database containing both active and decoy compounds (Chen et al. 2006; Huang et al. 2006). The active compounds in this study are the co-crystallized ligands that are either antagonists (9JU) or agonists (E3R & 5IW) of the CB2 receptor active site. The two main disadvantages of the EF include its dependence on the ratio of actives within the dataset and that it only gives an indication of the ability to identify active compounds and does not measure the ability to disregard decoys (Triballeau et al. 2005). Regardless, the EF is still widely used in the field of computer aided drug design and is effective in comparing the relative accuracy of different docking/scoring combinations when using the same decoy dataset (Triballeau et al. 2005).

Based on the data obtained from the RMSD validation, only the 6KPC crystal structure was used for both the EF and ROC curve validation. When comparing the EF^10%^ results to one another, the LibDock docking algorithm had the highest EF^10%^ value, i.e., 10.1, when combined with the PLP 1 & 2, PMF and LibDock Score scoring functions (Table 3). Similarly, the LigandFit docking algorithm had the highest EF^10%^ value, i.e., 10, when combined with the PLP 1 & 2, Jain and PMF scoring functions.

The ROC curve is another parameter used to express the success of a docking program in differentiating between active and decoy compounds and is preferred by some above the EF because it determines the absolute accuracy rather than the relative accuracy, which makes it possible to compare results between different studies (Triballeau et al. 2005). When combining LibDock with the PLP1 & 2, Jain, PMF, PMF 4 and the LibDock Score scoring functions, a ROC-AUC of ≤ 0.9 was obtained, which is considered excellent (Table3). Likewise, when combining LigandFit with the PLP 1 & 2, Jain, PMF and PMF 4 scoring functions, a ROC-AUC of ≤ 0.9 was obtained.

**Table 3:** EF^10%^ and ROC-AUC results for LibDock and LigandFit

Scoring function	Docking function			
	LibDock		LigandFit	
	EF^10%^†	ROC-AUC	EF^10%^‡	ROC-AUC
Native§	10.1	1.00±0.00**	0.0	0.57±0.12
LigScore 1	3.4	0.34±0.25	3.3	0.44±0.23
LigScore 2	3.4	0.39±0.25	3.3	0.65±0.15
PLP 1	10.1	1.00±0.00**	10.0	1.00±0.00**
PLP 2	10.1	1.00±0.00**	10.0	1.00±0.00**
Jain	6.7	0.94±0.05**	10.0	0.98±0.00**
PMF	10.1	1.00±0.00**	10.0	1.00±0.00**
PMF 4	6.7	0.92±0.04*	6.7	0.92±0.00*

* *p* < 0.05, ** *p* < 0.01

Native scoring function: LibDock (LibDock Score) & LigandFit (Dock Score)

† LibDock had a maximum EF of 101 (303 total compounds/3 active compounds)

‡ LigandFit had a maximum EF of 90.3 (271 total compounds/3 active compounds)

In conclusion, when paired with the correct scoring functions both LibDock and LigandFit were capable of accurately identifying the active co-crystallized ligands within a database also containing decoy compounds. These combinations can thus be used to predict, to an acceptable degree of accuracy, whether compounds have affinity and therefore might bind to the CB2 receptor to illicit a pharmacological action.

### Virtual screening

**Fig. 3:**
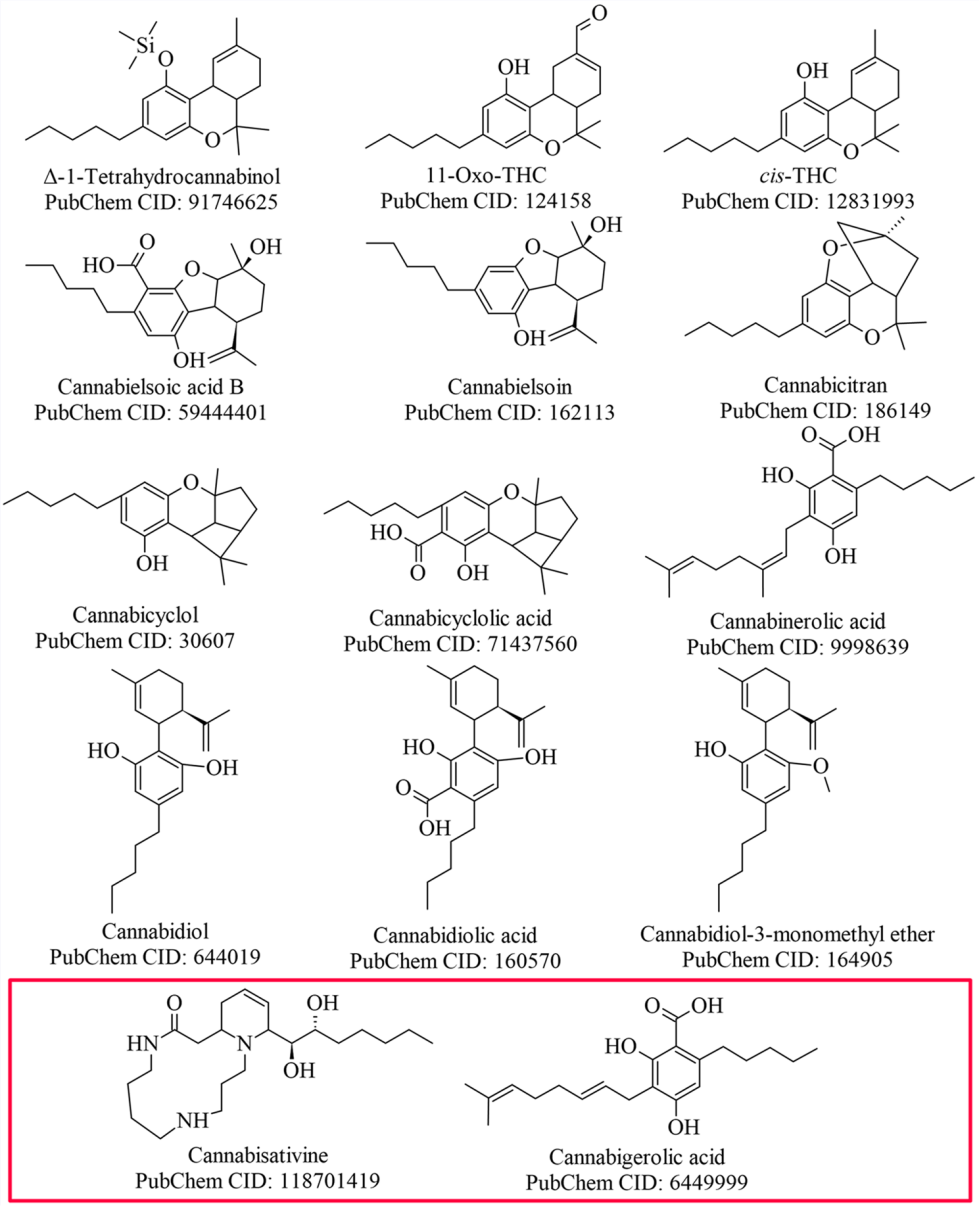
*Cannabis sativa* phytochemicals identified by LibDock and/or LigandFit to have affinity for the CB2 receptor

A total of 65 compounds, commonly found in *C. sativa*, were screened by means of docking to measure affinity for the CB2 receptor binding site (6KPC) with both LibDock and LigandFit and re-scored with the scoring functions that showed accurate results as determined during the validation described in the previous section. To increase the probability of only selecting compounds that would target the CB2 receptor, the most stringent ROC-AUC cut off was selected. *C. sativa* phytochemical compounds identified by LigandFit and/or LibDock are listed in the supplementary data as Table S1. From these phytochemicals that showed affinity for the CB2 receptor, four compounds were selected and procured for further evaluation in this study namely CBGA, cannabicyclol, CBDA, cannabicitran and cannabielsoin (Fig. 3). These compounds were selected based on availability at the time and served as proof of concept for potential nasal delivery of cannabinoids.

### LC-MS and fluorometric analytical method validation

All developed LC-MS methods were validated to confirm the range, linearity, precision, limit of detection and limit of quantification. All methods developed conformed to the following parameters: linearity in the ranges of 0.98–500.00 ng/mL with a minimum regression (R^2^) of 0.999, inter and intra-day precision with a maximum relative standard deviation (RSD) of 15% or less (Supplementary data, Table S2).

In summary, acyclovir had a R ^2^ of > 0.999 in the range of 0.98–500.00 ng/mL, an intraday precision RSD of 10.44% and an inter day precision RSD of 10.74%. Atenolol had a R ^2^ of > 0.999 in the range of 0.98–500.00 ng/mL, an intraday precision RSD of 4.37% and an inter day precision RSD of 7.75%. Cannabielsoin had a R ^2^ of > 0.999 in the range of 0.1–100 ng/mL, an intraday precision RSD of 5.19% and an inter day precision RSD of 1.01%. CBGA, CBDA, cannabicyclol and cannabicitran had a R ^2^ of > 0.999 in the range of 0.98–1000 ng/mL, an intraday precision RSD of 2.39, 8.26%, 2.41% and 5.08%, respectively and an inter day precision RSD of 4.48%, 4.13%, 6.62% and 7.40%, respectively.

The previously published fluorometric method for the quantification of LY was verified with regard to linearity within a range of 0.19 to 50.00 µg/mL (Rozehnal et al. 2012).

### Selected cannabinoid compound content assay in extract

The percentage content of each of the selected individual cannabinoid compounds was determined to be 0.399% w/v CBDA, 0.757% w/v cannabielsoin, 3.465% w/v cannabicyclol and 20.690% w/v cannabicitran. CBGA was omitted from further investigation as the content was too low (< 0.002% w/v).

After consideration of the percentage content and limit of quantification of each compound, working solutions of 20 mg/mL were prepared by dissolving the *C. sativa* extract in olive oil for the ex vivo permeation study.

### Membrane integrity test and permeation studies

A summary of the physicochemical properties, apparent permeability coefficient (*P*_app_) values, average percentage permeation and content in the lysed tissue at the end of the permeation study is given in Table 4.

In the membrane integrity study, LY exhibited an average apparent permeability coefficient (*P*_app_) value of 2.74 × 10^− 6^ cm/s across the excised sheep nasal epithelial tissues when KRBB was used as permeation liquid in both the donor and acceptor chambers. The *P*_app_ value for LY was 2.92 × 10^− 6^ cm/s when 0.5% v/v DMSO was added to KRBB in the acceptor chamber. These relatively low LY *P*_app_ values confirmed the integrity of the mounted excised sheep nasal epithelial tissue over the entire permeation study period (Gerber 2022).

The model drugs, acyclovir and atenolol, presented with *P*_app_ values of 12.4 × 10^− 6^ cm/s and 47.3 × 10^− 6^ cm/s, respectively. Acyclovir presented relatively low to moderate membrane permeability and atenolol presented moderate to high membrane permeability. Lucifer yellow was also used as a reference model compound that presented low nasal epithelial membrane permeability (in addition to indicate membrane integrity). The results obtained for these model drugs were used as benchmark regarding the rate and extent of permeation for each selected cannabinoid compound.

Cannabicyclol and cannabicitran exhibited *P*_app_ values of 8.51 × 10^− 6^ cm/s and 12.5 × 10^− 6^ cm/s, respectively, which are similar to that of acyclovir (*P*_app_ value of 12.4 × 10^− 6^ cm/s) indicating low to moderate membrane permeation (Table 4). CBDA presented with a *P*_app_ value of 3.8 × 10^− 6^ cm/s, which indicated low membrane permeation similar to that of LY. Cannabielsoin showed almost undetectable permeation with the extremely low *P*_app_ value of 3.26 × 10^− 8^ cm/s, which is markedly lower than the permeability of LY (*P*_app_ value of 2.92 × 10^− 6^ cm/s).

Comparison of the molecular mass of each selected cannabinoid compound revealed that the two cannabinoid compounds with the lowest molecular weights achieved the highest membrane permeation (cannabicyclol and cannabicitran, both 314.5 g/mol), which is in line with previous correlation between molecular weight and membrane permeation. CBDA with a molecular weight of 358.5 g/mol achieved 3.56% permeation, whereas cannabielsoin with a slightly lower molecular weight of 330.5 g/mol achieved an extremely low permeation percentage of 0.03%. The difference in the extent of membrane permeation between CBDA and cannabicyclol may be explained by the content of each compound within the cannabinoid extract namely 0.4% & 3.5%, respectively (Table 4).

**Table 4:** Summary of the permeability of the selected cannabinoid compounds and model drugs across excised ovine respiratory nasal epithelial tissue

Compound	Molecular weight (g/mol)	Log *P* value	Content in extract (%)	Content in lysed tissue (%)	Permeation (%)	*P*_app_ value
Cannabielsoin	330.468	5.4	0.757	1.094	0.028 ± 0.006	3.26 x 10^−8^
LY: KRBB	444.240	2.1	-	-	2.172 ± 1.284	2.74 x 10^−6^
LY: 0.5% DMSO in KRBB			-	-	2.267 ± 1.136	2.92 x 10^−6^
CBDA	358.500	6.6	0.399	0.288	3.560 ± 0.558	3.80 x 10^−6^
Cannabicyclol	314.470	6.0	3.465	0.296	7.546 ± 0.933	8.51 x 10^−6^
Acyclovir	225.210	−1.9	-	-	8.527 ± 2.394	1.24 x 10^−5^
Cannabicitran	314.470	5.9	20.690	0.166	11.054 ± 3.39	1.25 x 10^−5^
Atenolol	266.366	0.2	-	-	34.758 ± 7.409	4.73 x 10^−5^

*Molecular weight and Log *P* values obtained from Pubchem^®^

*Permeation and *P*_app_ values are given as a mean value from 6 diffusion cells

## Discussion

In silico docking was implemented as a virtual-screening step to assist in the filtering and prioritization of potentially active compounds for experimental screening. Docking generates ligand poses that resemble conformations previously confirmed with experimental crystallographic experiments. It cannot accurately determine in vitro binding affinities (Warren et al. 2006). Therefore, follow-up studies should further investigate the efficacy and pharmacological mechanisms of these compounds in the treatment of migraine.

Published data on the pharmacological activity of the four investigated compounds are limited. Early research in rabbits demonstrated that cannabicitran and cannabicyclol present with moderate, and cannabielsoin with slight, intraocular pressure lowering properties in rabbits (Elsohly et al. 1984). In addition, cannabicyclol expressed antiviral activity against Wuhan SARS-CoV-2 (Classen et al. 2024) and acts as a positive allosteric modulator for 5-HT1A receptors (Haghdoost et al. 2024). CBDA has been found to reduce nausea and vomiting (Rock et al. 2021) which could pose a potential benefit for migraineurs.

Despite the highly lipophilic nature of the cannabinoid compounds, the membrane permeation studies revealed that some of these compounds may be delivered across nasal epithelial tissue. It is of note that CBDA comprising of merely 0.4% w/v of the total cannabinoid extract achieved a permeation percentage of 3.56%. Cannabicyclol and cannabicitran presented with modest transport percentages of 7.55 and 11.05%, respectively. This indicated that these compounds with CB2 receptor affinity can possibly be delivered intranasally by direct nose-to-brain delivery or indirectly by delivery into the systemic circulation and then into the brain across the BBB. Although nasal epithelial tissue has been used to represent both systemic and direct nose-to-brain drug delivery (Salade et al. 2019), further studies are needed to distinguish between the permeation of the selected cannabinoid compounds across respiratory and olfactory nasal epithelial tissues.

Since a clear correlation between percentage content within the extract, molecular weight, log *P* values and the extent of membrane permeation could not be established, it may indicate that passive diffusion is possibly not the only mechanism of transport. It is recommended that future membrane permeation studies investigate the role of active uptake transporters, as well as efflux transporters, on the membrane permeation of these cannabinoid compounds. Another consideration is the possible influence of other phytochemical compounds present within the extract on the membrane permeation of selected cannabinoid compounds.

The present study was conducted using an unformulated crude extract to measure permeation of the selected cannabinoids. However, to improve the relatively low permeation of the cannabinoids, future studies should include the incorporation of formulation strategies such as nanoparticles, or addition of permeation enhancers such as chitosan.

## Conclusion

In silico docking procedures were successfully validated for the CB2 receptor and used to predict the binding affinity of common *C. sativa* phytochemicals. Compounds with acceptable docking scores (which represent acceptable receptor affinity) were identified namely cannabielsoin, CBDA, cannabicyclol and cannabicitran, which were selected for further investigation and evaluation of permeation across excised ovine nasal epithelial tissue. The compounds identified and evaluated for respiratory permeation during this study have shown potential in migraine treatment through binding to CB2 receptor and also showed potential to be delivered into the brain by means of nasal administration.

## Supplementary Information

Supplementary Material 1

## Data Availability

Data is provided within the manuscript or supplementary information files.
