# Supplementary material for: In silico receptor binding and ex vivo nasal epithelial membrane permeation studies of selected phytocannabinoids
Source: J Cannabis Res. 2026 Apr 16;8:56. doi: 10.1186/s42238-025-00363-y (PMC13104410; doi:10.1186/s42238-025-00363-y)
Supplement: Supplementary file 1 — Supplementary Material 1 [file 42238_2025_363_MOESM1_ESM.docx]

**Supplementary data: Table S1: Docking scores of *Cannabis sativa* phytochemicals**

| **PubChem ID** | **LigandFit** | | | | | **LibDock** | | | | | |
| --- | --- | --- | --- | --- | --- | --- | --- | --- | --- | --- | --- |
|  | **PLP 1** | **PLP 2** | **Jain** | **PMF** | **PMF 4** | **Native** | **PLP 1** | **PLP 2** | **Jain** | **PMF** | **PMF 4** |
| **10043** | - | - | - | - | - | 41.4 | 33.9 | 34.5 | 1.5 | 46.3 | 42.5 |
| **11110322** | 85.2 | 83.0 | 3.8 | 220.4 | 98.4 | 117.4 | 100.3 | 99.9 | 5.6 | 203.5 | 99.1 |
| **11551346** | 85.4 | 82.5 | 4.9 | 204.4 | 96.1 | 98.2 | 91.8 | 92.7 | 6.5 | 204.1 | 93.5 |
| **11601669** | 78.7 | 75.7 | 4.5 | 187.3 | 80.7 | 106.7 | 86.3 | 96.4 | 5.7 | 191.9 | 89.2 |
| **118701419** | 95.2 | 92.7 | 8.3* | 214.6 | 91.4 | 130.6 | 110.7 | 114.1 | 8.7* | 213.6 | 98.4 |
| **124158** | 95.7 | 91.6 | 5.5 | 216.8 | 104.0 | 117.8 | 101.8 | 97.8 | 6.9* | 205.4 | 98.3 |
| **12831993** | 87.3 | 84.0 | 5.2 | 213.3 | 97.5 | 112.0 | 94.9 | 93.0 | 7.1* | 210.8 | 95.4 |
| **145742** | - | - | - | - | - | 56.3 | 50.0 | 51.2 | 2.8 | 76.2 | 36.7 |
| **156460** | 93.0 | 90.5 | 4.0 | 226.6 | 104.1 | 118.5 | 96.3 | 101.6 | 6.7 | 226.4 | 106.0 |
| **160570** | 98.1 | 95.2 | 5.4 | 235.0 | 107.5 | 123.8 | 99.5 | 101.7 | 6.5 | 226.7 | 112.9* |
| **162113** | 81.3 | 77.2 | 4.8 | 228.7 | 103.1 | 116.2 | 98.2 | 94.7 | 7.1* | 211.4 | 96.6 |
| **164905** | 86.7 | 86.6 | 5.5 | 213.7 | 96.5 | 121.0 | 100.4 | 98.7 | 7.5* | 211.0 | 95.6 |
| **186149** | 82.3 | 75.9 | 4.4 | 215.6 | 100.4 | 110.8 | 90.6 | 90.5 | 8.8* | 213.5 | 95.7 |
| **192007** | 91.7 | 90.5 | 4.8 | 228.8 | 106.6 | 118.3 | 99.8 | 96.1 | 6.2 | 225.1 | 107.4 |
| **20586765** | 73.0 | 70.2 | 3.4 | 173.4 | 76.0 | 93.0 | 75.3 | 83.6 | 4.5 | 163.5 | 69.9 |
| **22805649** | 66.4 | 61.4 | 3.3 | 166.3 | 74.7 | 89.9 | 71.9 | 78.1 | 5.7 | 168.2 | 78.7 |
| **25105340** | 83.1 | 82.7 | 5.4 | 204.4 | 94.2 | 119.4 | 96.0 | 100.6 | 6.6 | 207.6 | 101.4 |
| **2543** | 88.3 | 83.8 | 4.2 | 208.7 | 95.8 | 116.5 | 94.8 | 95.0 | 6.7 | 204.2 | 92.8 |
| **305** | - | - | - | - | - | 52.3 | 45.2 | 49.8 | 3.2 | 54.6 | 50.8 |
| **30607** | 78.1 | 74.9 | 4.8 | 217.1 | 101.7 | 114.9 | 97.8 | 95.3 | 8.1* | 204.8 | 91.3 |
| **3081990** | 94.3 | 89.1 | 3.8 | 226.6 | 108.8 | 127.7 | 107.9 | 104.8 | 5.6 | 216.6 | 107.3 |
| **3084339** | 98.0 | 95.8 | 6.0 | 233.1 | 108.5 | 130.1 | 110.0 | 109.4 | 6.8 | 220.6 | 102.0 |
| **31268** | - | - | - | - | - | 24.9 | 20.0 | 20.3 | 0.8 | 45.7 | 17.1 |
| **449093** | 62.2 | 57.1 | 1.1 | 125.4 | 77.3 | 92.5 | 77.9 | 76.9 | 3.9 | 125.5 | 85.5 |
| **5315659** | 91.4 | 90.2 | 5.0 | 211.9 | 105.0 | 121.3 | 103.5 | 103.4 | 6.2 | 203.4 | 104.6 |
| **5570** | - | - | - | - | - | 63.6 | 54.6 | 53.0 | 2.5 | 76.3 | 53.7 |
| **59444381** | 79.8 | 77.7 | 3.9 | 200.5 | 97.7 | 108.7 | 96.9 | 98.0 | 6.7 | 193.5 | 95.6 |
| **59444383** | 95.9 | 92.4 | 4.1 | 220.4 | 108.7 | 122.1 | 106.2 | 104.7 | 6.0 | 213.7 | 108.0 |
| **59444387** | 82.7 | 80.1 | 4.2 | 210.8 | 93.6 | 116.7 | 93.2 | 94.9 | 5.5 | 205.0 | 90.4 |
| **59444391** | 98.7 | 94.1 | 4.5 | 226.5 | 108.7 | 130.3 | 110.8 | 108.6 | 6.1 | 225.5 | 106.5 |
| **59444401** | 81.3 | 81.2 | 5.5 | 230.2 | 106.6 | 129.1 | 110.4 | 114.5 | 7.1* | 243.6* | 120.0* |
| **59444405** | 82.4 | 82.5 | 5.5 | 230.5 | 105.7 | 124.3 | 109.5 | 104.0 | 5.8 | 230.1 | 106.1 |
| **59444407** | 86.1 | 82.6 | 3.9 | 196.5 | 99.1 | 114.8 | 95.4 | 100.5 | 5.9 | 185.3 | 94.7 |
| **59444416** | 83.3 | 78.7 | 3.5 | 210.4 | 92.7 | 112.1 | 90.1 | 87.6 | 5.8 | 200.6 | 88.5 |
| **595** | 59.0 | 61.1 | 0.8 | 130.6 | 79.2 | 84.6 | 72.9 | 77.0 | 2.5 | 110.0 | 75.8 |
| **5951** | - | - | - | - | - | 49.6 | 43.0 | 43.0 | 0.7 | 53.8 | 31.5 |
| **5962** | - | - | - | - | - | 69.6 | 63.0 | 66.8 | 3.8 | 93.0 | 49.3 |
| **6057** | - | - | - | - | - | 78.8 | 67.0 | 71.5 | 2.3 | 118.1 | 55.2 |
| **6106** | - | - | - | - | - | 59.9 | 52.7 | 57.6 | 2.0 | 90.4 | 48.3 |
| **6137** | - | - | - | - | - | 61.7 | 56.1 | 60.1 | 1.6 | 77.2 | 47.6 |
| **6140** | - | - | - | - | - | 81.0 | 74.2 | 75.6 | 2.4 | 114.8 | 64.2 |
| **622545** | 73.5 | 71.0 | 3.3 | 192.0 | 88.9 | 107.6 | 89.6 | 95.5 | 6.2 | 183.5 | 84.0 |
| **6274** | - | - | - | - | - | 70.1 | 66.3 | 67.9 | 2.2 | 90.1 | 63.6 |
| **628150** | 94.7 | 91.6 | 5.2 | 211.9 | 98.1 | 118.5 | 101.7 | 96.5 | 6.8 | 204.8 | 95.4 |
| **6287** | - | - | - | - | - | 54.7 | 48.3 | 49.2 | 1.5 | 78.0 | 39.9 |
| **6288** | - | - | - | - | - | 52.3 | 40.3 | 42.1 | 1.0 | 59.0 | 32.9 |
| **6305** | - | - | - | - | - | 94.3 | 78.3 | 85.4 | 3.6 | 127.3 | 74.9 |
| **6306** | - | - | - | - | - | 59.3 | 55.4 | 53.7 | 2.4 | 89.4 | 44.4 |
| **6322** | - | - | - | - | - | 80.4 | 69.9 | 71.8 | 3.5 | 108.8 | 58.8 |
| **638026** | 88.6 | 83.9 | 3.6 | 210.8 | 95.6 | 114.2 | 95.9 | 92.9 | 6.7 | 204.0 | 94.9 |
| **644019** | 85.0 | 82.6 | 5.1 | 209.7 | 93.5 | 113.2 | 95.9 | 96.1 | 7.0* | 205.0 | 97.1 |
| **6449999** | 104.7 | 102.3 | 5.2 | 235.9 | 117.2* | 134.1 | 113.1 | 108.2 | 6.3 | 223.5 | 114.4* |
| **6451726** | 76.5 | 78.5 | 4.6 | 183.1 | 83.1 | 106.7 | 89.7 | 93.9 | 5.5 | 176.3 | 88.3 |
| **6558** | - | - | - | - | - | 38.1 | 34.0 | 33.6 | 1.9 | 53.2 | 32.9 |
| **68313** | - | - | - | - | - | 76.5 | 65.9 | 66.5 | 3.2 | 105.2 | 63.8 |
| **71437560** | 78.1 | 79.4 | 5.4 | 230.8 | 108.2 | 120.3 | 102.6 | 99.6 | 8.8* | 216.4 | 103.6 |
| **7852** | - | - | - | - | - | 27.7 | 18.6 | 19.3 | 0.6 | 18.5 | 3.4 |
| **8007** | - | - | - | - | - | 39.4 | 35.5 | 36.0 | 1.7 | 52.6 | 35.0 |
| **8082** | - | - | - | - | - | 44.2 | 37.9 | 37.6 | 2.6 | 55.7 | 30.5 |
| **91746625** | - | - | - | - | - | 110.1 | 91.5 | 94.0 | 8.2* | 194.5 | 83.7 |
| **9308** | - | - | - | - | - | 76.3 | 66.0 | 64.6 | 3.7 | 94.4 | 66.2 |
| **93147** | 77.0 | 71.8 | 3.1 | 192.2 | 89.6 | 101.1 | 82.3 | 79.9 | 6.3 | 191.4 | 84.6 |
| **98523** | 97.5 | 92.8 | 4.3 | 225.8 | 108.2 | 127.2 | 105.1 | 104.0 | 5.6 | 220.8 | 105.6 |
| **9966466** | 84.4 | 81.8 | 4.0 | 198.5 | 101.4 | 111.5 | 92.7 | 94.0 | 6.4 | 188.8 | 100.5 |
| **9998639** | 99.8 | 95.6 | 4.6 | 231.5 | 107.6 | 132.8 | 119.2 | 117.3 | 6.6 | 228.2 | 110.9* |

Note: If there is no data for a specific compound, that compound could not be docked and was rejected by the relevant docking function.

Note: The ROC curve cut-off values where 100% of the active compounds were identified were as follows; LigandFit; PLP 1 (107.6), PLP- 2 (103.1), Jain (6.5), PMF (242.2), PMF 4 (112.6).

Note: The ROC curve cut-off values where 100% of the active compounds were identified were as follows; LibDock; Native (142.5), PLP 1 (120.7), PLP 2 (118.0), Jain (6.9), PMF (238.0), PMF 4 (110.4).

* Is considered active when using a ROC curve cut-off of 100.

**Supplementary data: Table S2: Summary of validation results and parameter values for all LC-MS analytical methods**

|  |  | Range | Linearity | Inter Day Precision | | | Intra Day Precision | | | LOD | | LOQ |
| --- | --- | --- | --- | --- | --- | --- | --- | --- | --- | --- | --- | --- |
|  |  | Correlation Coefficient (R^2^) | | % Relative standard deviation (RSD) | | | | | | Concentration  (ng/mL) | | |
| Cannabielsoin | Concentration (ng/mL) | 0.1 - 100 | 0.1 - 100 | 3.13 | 12.50 | 50.00 | 3.13 | 12.50 | 50.00 | | 0.01 | 0.04 |
|  | Parameter value | 0.999 | 0.999 | 0.98 | 0.86 | 1.01 | 4.85 | 5.19 | 4.50 | |  |  |
| Cannabidiolic acid | Concentration (ng/mL) | 0.98 - 1000 | 0.98 - 1000 | 7.81 | 31.25 | 125.00 | 7.81 | 31.25 | 125.00 | | 1.01 | 3.07 |
|  | Parameter value | 0.999 | 0.999 | 4.13 | 2.56 | 2.50 | 8.26 | 2.67 | 1.13 | |  |  |
| Cannabicyclol | Concentration (ng/mL) | 0.98 - 1000 | 0.98 - 1000 | 7.81 | 31.25 | 125.00 | 7.81 | 31.25 | 125.00 | | 0.51 | 0.90 |
|  | Parameter value | 0.999 | 0.999 | 6.62 | 5.37 | 4.99 | 2.41 | 1.27 | 0.63 | |  |  |
| Cannabicitran | Concentration (ng/mL) | 0.98 - 1000 | 0.98 - 1000 | 7.81 | 31.25 | 125.00 | 7.81 | 31.25 | 125.00 | | 0.26 | 0.78 |
|  | Parameter value | 0.999 | 0.999 | 7.40 | 6.08 | 6.27 | 5.08 | 2.03 | 1.30 | |  |  |
| Acyclovir | Concentration (ng/mL) | 0.98 - 500 | 0.98 - 500 | 1.95 | 31.25 | 500.00 | 1.95 | 31.25 | 500.00 | | 10.52 | 31.87 |
|  | Parameter value | 0.999 | 0.999 | 10.85 | 10.74 | 9.60 | 3.20 | 10.44 | 9.06 | |  |  |
| Atenolol | Concentration (ng/mL) | 0.98 - 500 | 0.98 - 500 | 7.81 | 31.25 | 125.00 | 7.81 | 31.25 | 125.00 | | 5.66 | 17.16 |
|  | Parameter value | 0.999 | 0.999 | 7.75 | 6.01 | 4.88 | 2.79 | 4.37 | 1.34 | |  |  |
